# Psychological Distress and Problematic Mobile Phone Use Among Adolescents: The Mediating Role of Maladaptive Cognitions and the Moderating Role of Effortful Control

**DOI:** 10.3389/fpsyg.2019.01589

**Published:** 2019-07-12

**Authors:** Ru-De Liu, Wei Hong, Yi Ding, Tian Po Oei, Rui Zhen, Shuyang Jiang, Jingxuan Liu

**Affiliations:** ^1^Beijing Key Laboratory of Applied Experimental Psychology, National Demonstration Center for Experimental Psychology Education, Faculty of Psychology, Beijing Normal University, Beijing, China; ^2^Graduate School of Education, Fordham University, New York, NY, United States; ^3^School of Psychology, University of Queensland, St Lucia, QLD, Australia; ^4^Department of Psychology, James Cook University, Singapore, Singapore; ^5^Institute of Psychological Sciences, Hangzhou Normal University, Hangzhou, China; ^6^Trinity School of Art and Science, Duke University, Durham, NC, United States

**Keywords:** psychological distress, problematic mobile phone use, maladaptive cognitions, effortful control, adolescents, moderated mediation

## Abstract

Previous studies have documented that psychological distress is related to problematic mobile phone use (PMPU), and sporadic research has investigated the potential mechanisms underlying the association. Based on the cognitive-behavioral model of pathological Internet use (PIU), the self-control theory, and the problem-behavior theory, this study aimed to examine the mediating role of maladaptive cognitions toward mobile phones and the moderating role of effortful control between psychological distress and PMPU. Data were collected from 1,799 secondary school students (45.1% male; *M*_age_ = 14.07, SD = 1.69) using four self-reported questionnaires. The results revealed that maladaptive cognitions toward mobile phones partially mediated the relationship between psychological distress and PMPU. In addition, effortful control as a protective factor attenuated the indirect effect from psychological distress to PMPU. These findings advanced the understanding of the etiology of PMPU and the need to develop effective strategies for prevention, suggesting that schools and families should pay additional attention to students with psychological distress. Targeted interventions for integrating online and offline worlds and effortful control training programs may help to prevent adolescents from engaging in PMPU.

## Introduction

Information technology is developing rapidly across the globe, and people are surrounded by manifold electronic devices. Mobile phones enhance convenience, convey information, and provide entertainment (Elhai et al., 2017; Castellacci and Tveito, 2018); thus, they have penetrated into every aspect of people’s daily lives, increasing the potential for dependency on mobile phones, especially among adolescents. Mobile phone overuse behaviors associated with addiction-like symptoms, such as craving, withdrawal, and loss of control, have been described as problematic mobile phone use (PMPU; Foerster et al., 2015; Chun, 2018). According to an investigation among British adolescents, 10% of participants were problematic mobile phone users and 20.5% were potential problematic users. Adolescents aged 11–14 were subject to higher risks of PMPU than other age groups (Lopez-Fernandez et al., 2014). Research has found that PMPU can lead to a multitude of negative outcomes, including sleep disturbances (Thomée et al., 2011), academic failure (Lepp et al., 2014), interpersonal distress (Seo et al., 2016), internalizing symptoms (Marengo et al., 2018), and poor mental health status (Chen et al., 2016).

Such hazards have captured public concern, and much importance has been placed on determining the possible antecedents of PMPU. Researchers have attempted to explore the developmental components of PMPU, mainly personal factors, such as anxiety, depression, low self-esteem, and self-control (Hou et al., 2018), and environmental attributes, such as family context and school climate (Badenes-Ribera et al., 2019), as antecedents of PMPU (Anderson et al., 2017; Fumero et al., 2018; Martin-Perpina et al., 2019). Targeted interventions, such as education on how to cope with negative emotions and unpleasant states and training in self-awareness and self-regulation related to mobile phone use, are reported to be of value in reducing and even overcoming mobile phone addiction (Chun, 2018). Although investigation of high-risk factors of PMPU, particularly psychopathological factors, has garnered considerable empirical support, the underlying mechanisms (i.e., what works for whom) have not been rigorously evaluated. To address the gap, Davis (2001) suggested that internal cognitive factors and individual differences in self-control were linked. Therefore, the current study proposed to examine whether the relationship between psychological distress and PMPU could be mediated by maladaptive cognitions toward mobile phones and whether the mediation of maladaptive cognitions could be moderated by effortful control among adolescents.

### Psychological Distress and PMPU

Psychological distress refers to general but uncomfortable feelings of anxiety, depression, and emotional upset and reflects an internal state of mental health (Bore et al., 2016). Researchers have argued that it serves as a potential precursor to PMPU (Anderson et al., 2017). Based on the self-medication hypothesis of addiction, Park et al. (2014) held that people who suffer from psychological distress tend to cope with their negative emotions by seeking ways of self-soothing, such as interacting on mobile phones as a temporary remedy. Once being able to obtain relief from mobile phone interaction, people are more likely to regard the use of mobile phones as a helpful coping strategy, which can lead to automatic activation and potential dependence (Kim et al., 2015; Nicholls et al., 2015). A substantial body of research supported this notion and found that psychological distress is associated with pathological Internet use (PIU) among adolescents and emerging adults (Xiuqin et al., 2010; Lu et al., 2011; Yadav et al., 2013; Tang et al., 2014; Wong et al., 2014; Anand et al., 2018). Other researchers found that anxiety, depression, and stress severity positively predicted mobile phone addiction (Gao et al., 2018). Therefore, it is reasonable to postulate that psychological distress is closely associated with increased severity of PMPU among adolescents.

### Maladaptive Cognitions as a Mediator

Maladaptive cognitions, which refer to cognitive biases toward the self and the world, are argued to be the central mediating factors in the cognitive-behavioral model of PIU (Davis, 2001). Psychopathology (i.e., depression and anxiety) as a distal necessary cause affects PIU symptoms through maladaptive cognitions as proximal causes. Given that PMPU and PIU are highly overlapping and cannot be considered separately (Chin and Leung, 2018), the influence mechanism in the cognitive-behavioral model of PIU would also apply to the use of mobile phones. That is, maladaptive cognitions toward mobile phones can be hypothesized to mediate the relationship between psychological distress and PMPU.

In particular, psychological distress, such as negative emotional states in real life, makes people prone to feel less comfortable and/or valued, compared to situations experienced with mobile phones. This formation of distorted cognitions about the online and offline worlds likely increases the excessive use of mobile phones (Billieux, 2012). Discrepancies between the virtual and the actual “self” acted as a reliable predictor of dysfunctional use of online services (Billieux et al., 2015). Importantly, cognitive distortion has been shown to mediate the relationships between anxiety, depression, stress, and Internet addiction (Lu and Yeo, 2015; Teo et al., 2017). Longitudinal research also has found that psychosocial factors (e.g., shyness, loneliness, and interpersonal distress) predict maladaptive cognitions, which in turn intensify the severity of PIU (Tian et al., 2017). Thus, psychological distress has a potential effect on adolescents’ PMPU through maladaptive cognitions toward mobile phones.

### Effortful Control as a Moderator

Another essential factor underlying the association between psychological distress and PMPU is effortful control, which refers to the efficiency of executive attention, including activating relevant attention and suppressing inappropriate responses (Zhou et al., 2010). Effortful control has a wide range of synonyms, such as self-control, self-regulation, executive control, and ego strength, and shows profound benefits across every aspect of life functioning (Duckworth, 2011). Previous research found that effortful control was negatively associated with PIU (Özdemir et al., 2014; Anderson et al., 2017) and PMPU (Jiang and Zhao, 2017), which coincided with the findings from neurobiological studies (Dieter et al., 2017).

Importantly, drawing on the problem-behavior theory, psychosocial factors and personality characteristics interact and engender a dynamic state that results in problem behaviors (Jessor and Jessor, 1977). It is suggested that except for the potential impact of psychological distress, effort control (salient trait characteristics) may also have a modulating effect on the occurrences of PMPU (Billieux, 2012). In support of this, Davis (2001) posited that detrimental outcomes pertaining to Internet use would occur unless individuals exercised control, which indicated that when people are at higher levels of effortful control, the adverse effects of vulnerabilities on PMPU are likely to become weaker. Previous research strengthened this notion by showing that effortful control could moderate the relationship between low self-esteem (a negative aspect of cognitions toward self) and PIU (Park et al., 2014). In summary, effortful control as a protective resource likely attenuates the negative effect of maladaptive cognitions on PMPU among adolescents.

### The Present Study

Ample evidence has indicated that psychological distress is associated with PMPU, but the mechanism underlying this relationship has not been evaluated rigorously. Thus, the present study aimed to investigate how psychological distress impacts adolescents’ PMPU and when the predictive effect becomes stronger. Based on the self-medication hypothesis and the cognitive-behavioral model of PIU, combined with problem-behavior theory, the present study proposed that maladaptive cognitions toward mobile phones would play a mediating role in the association between psychological distress and PMPU (**H1**), and effortful control would have a moderating effect on the mediation of maladaptive cognitions (**H2**). Specifically, the potential impact of psychological distress on the severity of PMPU *via* maladaptive cognitions would become weaker when adolescents exhibited higher levels of effortful control.

## Materials and Methods

### Participants and Procedure

Participants were 1,799 students (33.5% seventh graders, 22.0% eighth graders, 21.7% 10th graders, and 22.8% 11th graders) aged 12–18 years (*M* = 14.07, SD = 1.69) who were recruited from secondary schools in Beijing, China. Among them, 812 were male (45.1%) and 927 were female (51.5%), and 60 did not report gender (3.3%). All participants reported having more than one Internet-accessible mobile phone and more than 1 year of mobile phone use experience.

For the present research, approval was obtained from the Academic Ethics Committee of the Faculty of Psychology at the Beijing Normal University. Before completing the formal survey, all participation-related individuals, including school principals, teachers, and students, gave their written informed consent, noting that they were aware that the investigation was anonymous and confidential and that participants had the right to withdraw at any time without explanation. The paper-and-pencil questionnaires were distributed by trained research assistants and collected in the regular classrooms. It took 20 min on average to complete the questionnaires.

### Measures

#### Psychological Distress

Psychological distress was measured by the Kessler Psychological Distress Scale (Andrews and Slade, 2001), which has been adapted and verified in the Chinese context (Zhou et al., 2008). The scale consists of two subordinate structures with 10 items, including anxiety (sample item, did you feel so nervous that nothing could calm you down) and depression (sample item, did you feel so sad that nothing could cheer you up). Participants responded to the items using a 5-point Likert scale, ranging from 1 (*none of the time*) to 5 (*all of the time*), with higher scores indicating a higher level of psychological distress. The results of confirmatory factor analysis (CFA) supported the two-dimension construct validity, *χ*^2^(32) = 368.90, CFI = 0.98, TLI = 0.97, RMSEA (90% CI) = 0.076 (0.070–0.084), SRMR = 0.025. Additionally, the internal reliability and convergent validity of this scale in the current study were satisfactory [Cronbach’s *α* = 0.94, the composite reliability (CR) = 0.94, the average variance extracted (AVE) = 0.61].

#### Maladaptive Cognitions

Maladaptive cognitions toward mobile phones were assessed by the Chinese Adolescents’ Maladaptive Cognitions Scale adapted from Mai et al. (2012). The scale was modified to fit the current study by changing the word “online” to “on mobile phones.” The scale has a three-factor structure with 12 items, including social comfort (sample item, when surfing on the mobile phone, I can say things that could never be said in real life), distraction (sample item, I do not have to think about the difficult homework when I am on the mobile phone), and self-realization (sample item, I feel myself more powerful when I am on the mobile phone). Participants responded to the items using a 5-point Likert scale from 1 (*completely disagree*) to 5 (*completely agree*), with higher scores indicating a higher level of maladaptive cognitions toward mobile phones. The results of CFA supported the three-dimension construct validity, *χ*^2^(45) = 466.89, CFI = 0.97, TLI = 0.96, RMSEA (90% CI) = 0.072 (0.066–0.078), SRMR = 0.037. Additionally, the scale in this study showed acceptable internal reliability and convergent validity (Cronbach’s *α* = 0.92, CR = 0.92, AVE = 0.48).

#### Effortful Control

Effortful control was measured by a Chinese version of a short form of the Early Adolescent Temperament Questionnaire-Revised (Li et al., 2016). The scale includes three aspects with 16 items, consisting of attention control (sample item, it is easy for me to fully concentrate on homework problems), activation control (sample item, if I have a hard assignment to do, I get started right away), and inhibitory control (sample item, I can stick with my plans and goals). Participants responded to each item with a 5-point Likert scale, ranging from 1 (*almost always untrue of you*) to 5 (*almost always true of you*), with higher scores indicating a higher level of effortful control. The results of CFA supported the three-dimension construct validity, *χ*^2^(74) = 590.42, CFI = 0.94, TLI = 0.90, RMSEA (90% CI) = 0.062 (0.058–0.067), SRMR = 0.048. Additionally, the internal reliability and convergent validity of this scale in the current study were acceptable (Cronbach’s *α* = 0.80, CR = 0.73, AVE = 0.37).

#### Problematic Mobile Phone Use

Problematic mobile phone use was assessed by a Chinese version of the short form of the Mobile Phone Problem Use Scale (Foerster et al., 2015; Hong et al., 2019). The scale comprises five aspects with 10 items, such as craving, withdrawal, peer dependence, loss of control, and negative life consequences. Adolescents rated to what extent they agreed with each description (sample item, I feel anxious if I have not checked for messages or switched on my mobile phone for some time). They responded to each item using a 5-point Likert scale, ranging from 1 (*strongly disagree*) to 5 (*strongly agree*), with higher scores indicating a higher level of problematic mobile phone use. The results of CFA supported the five-dimension construct validity, *χ*^2^(26) = 324.00, CFI = 0.96, TLI = 0.92, RMSEA (90% CI) = 0.080 (0.072–0.088), SRMR = 0.044. Additionally, the scale in this study showed adequate internal reliability and convergent validity (Cronbach’s *α* = 0.86, CR = 0.84, AVE = 0.57).

### Data Analyses

Means, standard deviations, and Pearson correlations for the levels of psychological distress, maladaptive cognitions, effortful control, and PMPU, together with demographic variables (e.g., gender and grade), were conducted using SPSS 19.0. Structural equation modeling (SEM) analyses were conducted to examine the mediating role of maladaptive cognitions and the moderating role of effortful control using Mplus 7.0. Missing data were handled using maximum likelihood estimation (ML). According to Wen et al. (2004), evaluating model fit depends on the following indexes: chi-square values (*χ*^2^), the comparative fit index (CFI), the Tucker-Lewis fit index (TLI), the root mean square error of approximation (RMSEA), and the standardized root-mean-square residual (SRMR). Notably, CFI and TLI values larger than 0.9 and RMSEA and SRMR values less than 0.08 indicate an acceptable model fit.

## Results

### Preliminary Analyses

Before performing formal analyses, we ensured that serious common method bias did not exist in these subjective self-reported data. According to Harman’s single factor test, the confirmatory factor analysis with one fixed factor showed that the model fit was as follows: *χ*^2^(1080) = 26798.91, CFI = 0.45, TLI = 0.43, RMSEA (90% CI) = 0.115 (0.114–0.116), SRMR = 0.116. Such unsatisfactory model fit indicated that the self-reported data did not have serious common method bias and were appropriate for further analyses (Zhou and Long, 2004).

Means, standard deviations, and correlations for the main variables are presented in Table 1. As shown, gender had a weak correlation with psychological distress (*r* = 0.07, *p* < 0.05) and PMPU (*r* = 0.05, *p* < 0.05). By contrast, grade was positively and significantly related to all the variables except for maladaptive cognitions (*r* = 0.04, *p* > 0.05), suggesting that grade should be incorporated into the model to control its effects. With regard to the main variables, there were moderate correlations among psychological distress, maladaptive cognitions, effortful control, and PMPU (|*r*| from 0.38 to 0.50, *p* < 0.01).

**Table 1:** Means, standard deviations and correlations.

	*M*	SD	1	2	3	4	5	6
1. Grade	8.78	1.63	–					
2. Gender	1.53	0.50	0.09^**^	–				
3. Psychological distress	2.37	0.96	0.16^**^	0.07^*^	–			
4. Maladaptive cognitions	2.76	0.87	0.04	−0.01	0.38^**^	–		
5. Effortful control	3.45	0.56	−0.19^**^	0.01	−0.44^**^	−0.40^**^	–	
6. Problematic mobile phone use	2.51	0.81	0.14^**^	0.05^*^	0.40^**^	0.44^**^	−0.50^**^	–

Gender (1 = male, 2 = female). ^*^p < 0.05, ^**^p < 0.01.

### Examining the Mediating Role of Maladaptive Cognitions

To test Hypothesis 1, a mediation model was used to examine whether maladaptive cognitions toward mobile phones could mediate the relationship between psychological distress and PMPU. First, the direct model showed a satisfactory model fit, *χ*^2^(19) = 198.39, CFI = 0.97, TLI = 0.95, RMSEA (90% CI) = 0.072 (0.064–0.082), SRMR = 0.047. The results revealed that psychological distress positively predicted PMPU after controlling for the effects of grade (*β* = 0.43, *p* < 0.001). Afterward, according to the SEM approach to examine mediating effects (Cheung, 2007), with maladaptive cognitions toward mobile phones incorporated, the mediation model showed an adequate model fit, *χ*^2^(41) = 398.08, CFI = 0.96, TLI = 0.94, RMSEA (90% CI) = 0.070 (0.063–0.076), SRMR = 0.047. Psychological distress not only positively predicted PMPU (*β* = 0.26, *p* < 0.001) but could also predict maladaptive cognitions (*β* = 0.43, *p* < 0.001), which in turn predicted PMPU (*β* = 0.39, *p* < 0.001). To further examine its effect, a bias-corrected bootstrap with 1,000 deriving samples was adopted; the results demonstrated that the 95% confidence interval of the indirect path coefficient ranged from 0.13 to 0.21 (not including zero indicates the significance). Therefore, maladaptive cognitions toward mobile phones were shown to partially mediate the association between psychological distress and PMPU, supporting Hypothesis 1.

### Examining the Moderated Mediation Model

**Figure 1:**
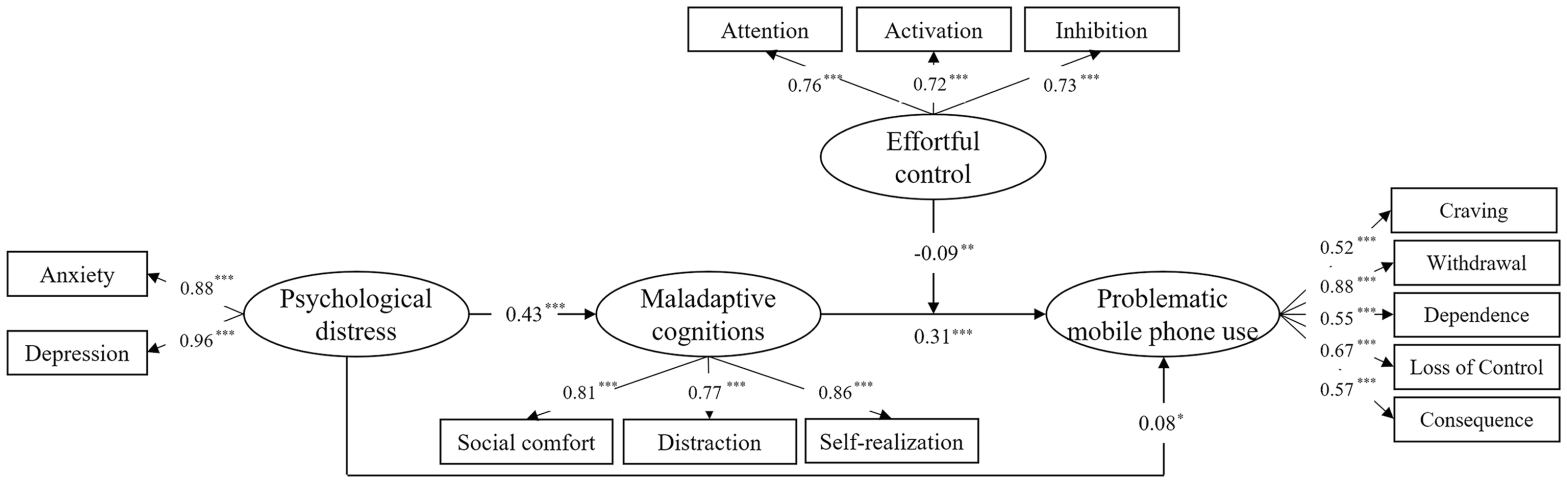
A moderated model after controlling for the effects of grade. All coefficient estimates are completely standardized. *Note.* ^*^p < 0.05, ^**^p < 0.01, ^***^p < 0.001.

After the mediation model had been tested, we examined whether effortful control could moderate the relationship between maladaptive cognitions toward mobile phones and PMPU. According to Wen and Ye (2014), after standardizing the variables, an interaction term (i.e., effortful control and maladaptive cognitions toward mobile phones) was produced and a moderated mediation model was formulated; the results showed an adequate model fit, *χ*^2^(111) = 957.00, CFI = 0.93, TLI = 0.91, RMSEA (90% CI) = 0.065 (0.061–0.069), SRMR = 0.065. As illustrated in Figure 1, psychological distress predicted maladaptive cognitions (*β* = 0.43, *p* < 0.001), which in turn predicted PMPU (*β* = 0.31, *p* < 0.001). Additionally, the interaction term of effortful control and maladaptive cognitions significantly predicted PMPU (*β* = −0.09, *p* < 0.01). Likewise, the bias-corrected bootstrap results revealed that the 95% confidence interval ranged from −0.10 to −0.02 (not including zero), which indicated that effortful control could moderate the indirect effect of psychological distress on PMPU through maladaptive cognitions.

**Figure 2:**
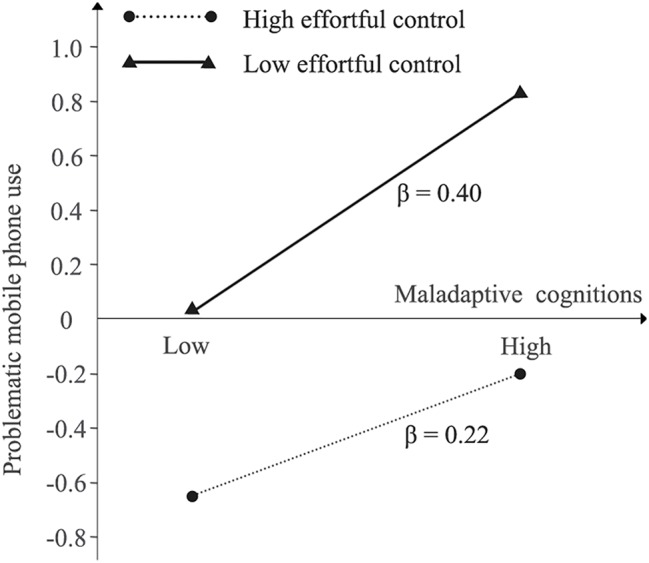
Problematic mobile phone use as a function of maladaptive cognitions and effortful control controlling for psychological distress.

To determine the essence of the interaction effect, a simple slope analysis was conducted. According to the levels of the moderator (i.e., effortful control), participants could be divided into high (i.e., *M* + SD) and low (i.e., *M* − SD) counterparts. The effects of maladaptive cognitions on PMPU were examined under different levels of effortful control after controlling for psychological distress. As shown in Figure 2, under a high level of effortful control, maladaptive cognitions predicted PMPU (simple slope = 0.22, *p* < 0.001). By contrast, under a low level of effortful control, maladaptive cognitions were also able to predict PMPU (simple slope = 0.40, *p* < 0.001). Additionally, Edwards and Lambert (2007) recommended further examination of the effect sizes of the indirect paths under different levels of the moderator. Similarly, under a high level of effortful control, the mediating effect size from psychological distress to PMPU through maladaptive cognitions ranged from 0.03 to 0.08 (95% CI). By comparison, under a low level, the mediating effect size increased, ranging from 0.07 to 0.13 (95% CI). In spite of the significance of both paths, a statistical difference was found by conducting a Wald test (*t* = −2.77, *p* < 0.01). Taken together, the results indicated that the conditional mediation from psychological distress to PMPU *via* maladaptive cognitions toward mobile phones would weaken with the increase in effortful control, thus supporting Hypothesis 2.

## Discussion

This study extended the previous research and verified the relationship between psychological distress and PMPU among adolescents (Kim et al., 2015; Gao et al., 2018). Also, it addressed the potential mechanisms underlying the association. With the moderated mediation model tested, the negative effects of psychological distress on PMPU were interpreted by maladaptive cognitions toward mobile phones as a potential mediator, and the second link of the indirect path was moderated by effortful control. These findings suggested that effortful control as a protective resource attenuated the adverse impacts of psychological distress on PMPU through distorted cognitions, which could help to advance the understanding of the etiology of problem behaviors related to mobile phones.

### Maladaptive Cognitions as a Mediator

The results identified the role that mobile phone-related maladaptive cognitions played in partially mediating the relationship between psychological distress and PMPU, which extended the cognitive-behavioral model in the context of the use of mobile phones (Davis, 2001). Consistent with previous research (Lu and Yeo, 2015; Teo et al., 2017), the results showed that psychological distress predicted the generation and maintenance of adolescents’ distorted cognitions toward mobile phones, which further increases the potential risk of PMPU.

In particular, people who suffer from anxiety and depression are likely to turn to a substitutable environment and participate in online activities (Park et al., 2014; Elhai et al., 2017). For instance, in the Chinese context, online communication services (e.g., WeChat, Tencent QQ) are most commonly used (CNNIC, 2019) because of their invisibility, anonymity, and the ability to reduce physical cues, which could allow people to feel less restrained and more comfortable online than offline (Weidman et al., 2012; Young and Lo, 2012). Additionally, people with anxiety, depression, and emotional upset can perceive social support from the online context (Hou et al., 2018; Lo, 2019). Such desirable experiences make it rewarding for adolescents to engage in the use of mobile phones and could lead to addiction-like symptoms.

Moreover, adolescents, especially Chinese students who confront intense competition for further education, tend to have high levels of academic pressure associated with psychological distress, such as anxiety about future. Dissociative imagination on mobile phones can separate users from stress and responsibilities (Suler, 2004), providing them with temporary escape from the unpleasant experiences (Settanni et al., 2018; Zhu et al., 2019). These feelings of relief that users are able to achieve possibly encourage increased use of mobile phones, thus reducing time and resources for face-to-face interaction and depriving them from the offline world, which increases the probability of problematic use (Li et al., 2010).

One more possible explanation is that mobile phones provide various platforms for self-presentation. For instance, psychologically distressed people who are troubled in real life may discover special talents from mobile phones (e.g., playing online games) that satisfy their sense of achievement, and such self-fulfillment is likely to reinforce their out-of-control use of mobile phones (Mai et al., 2012). In short, people with psychological distress appear to be more vulnerable to forming and maintaining maladaptive cognitions, such as *social comfort*, *distraction*, and *self-realization*, which in turn potentially exacerbates the severity of PMPU, especially among adolescents.

### Effortful Control as a Moderator

It is worth noting that effortful control functions as a buffer, which could attenuate the negative effect of the maladaptive cognitions on PMPU even though the users experienced psychological distress, as postulated by the self-control theory (Hagger et al., 2010) and the problem-behavior theory (Jessor and Jessor, 1977). People low in effortful control often exhibit more impulsiveness and are therefore more likely to use the Internet and mobile phones in maladaptive ways (Billieux, 2012; Anderson et al., 2017; Jiang and Zhao, 2017; Martin-Perpina et al., 2019). Consistent with previous studies that found effortful control could modulate the adverse impacts of cognitions about negative aspects of self (Park et al., 2014) and external environment risks (Li et al., 2016) of PIU, the results revealed that adolescents lacking effortful control, particularly inhibition control, were more vulnerable to the potential influence on PMPU when they experienced psychological distress and distorted cognitions. Conversely, when self-control capacity improved, people were more likely to overcome impulses and regulate behavior better, and then the negative impacts on PMPU would decrease (Hagger et al., 2010). In summary, these findings provided empirical evidence to support the notion that temperamental characteristics have a moderating effect on the influence of psychosocial distress on PMPU through maladaptive cognitions.

### Limitations

There are several limitations to the current study. First, the data primarily relied on self-reported information. Thus, response bias might have influenced the final results. Second, the cross-sectional design could not be used to make causal inferences. Future longitudinal research or experimental designs are needed to further ascertain the particular directions and relationships among these variables. Third, although the model fit indices of our hypothetical model were adequate, alternative models cannot be completely excluded (MaCcallum et al., 1993). Fourth, the average variance extracted from the subscale of the Early Adolescent Temperament Questionnaire-Revised in our study failed to reach the threshold of 0.5 (Fornell and Larcker, 1981); thus, future research could further strengthen the reliability and validity of this scale. Fifth, although questionnaires administrated by paper-and-pencil and online devices have no significant differences in psychometrics (Cai et al., 2008; Vallejo et al., 2008), it may be more desirable to use online questionnaires (e.g., through apps or websites on mobile phones) because doing so can control the quality of the data and avoid coding errors (Cook et al., 2001). Additionally, the present study only investigated Chinese secondary school students, and therefore, caution should be exercised before generalization to other groups, such as people in different developmental stages and/or from different cultural background. Adults, especially older adults, may adopt different distress coping strategies, such as alcohol consumption (Blumenthal et al., 2018), and thus may develop different problematic behaviors, including but not limited to PMPU. Finally, different schools may have different policies for managing students’ mobile phone usage, especially if the schools are of different nationalities or cultural backgrounds. Future studies are warranted to take these factors into consideration when exploring behaviors related to mobile phone use.

### Implications

Despite these limitations, the present study provided some theoretical and practical implications. Based on our literature review, it is one of the few research papers to incorporate psychosocial factors, cognitive factors, and personality traits into an integrated model. The findings extended the cognitive-behavioral model of PIU to mobile phone use. More importantly, with effortful control introduced, the results provided empirical evidence to identify how and when psychological distress exerts an influence on PMPU and advanced a better understanding of the etiology of addiction-like symptoms related to mobile phone use.

The findings of our study could be applied to the processes of prevention and intervention: Adolescents with psychological distress and maladaptive cognitions are at higher risk of developing PMPU, and more attention should be paid to them. Meanwhile, targeted interventions that inform instructions to constructively cope with life troubles and psychological distress and to harmoniously integrate online and offline worlds, including self-identify, social relationships and social functions, could be effective in helping vulnerable adolescents to use mobile phones appropriately (Lin et al., 2018). In addition, our findings pertaining to effortful control as an important protective factor that modulates the negative impacts of vulnerabilities could also contribute to preventing adolescents from engaging in PMPU. Even though effortful control is somewhat stable, it is also malleable and improvable, especially in the early developmental stages (Oaten and Cheng, 2006). Thus, schools and parents can educate students about the potential harms of obsessive mobile phone use and guide them to set self-reinforcing goals to monitor their frequency and duration of mobile phone use (Chun, 2018). These strategies may be instrumental in helping them to overcome impulses efficiently and to regulate themselves properly, promoting healthy ways of using mobile phones.

## Data Availability

The datasets generated for this study are available on request to the corresponding author.

## Ethics Statement

For the present research, approval was obtained from the Academic Ethics Committee of the Faculty of Psychology at the Beijing Normal University and the local education authorities. Before the formal survey, all participating-related objects, including school principals, teachers and students, gave their written informed consent, noting they were aware that the investigation was anonymous and confidential, and participants had the right to quit at any time without any explanation.

## Author Contributions

R-DL and WH designed the study, analyzed data, and drafted the manuscript. YD, TO, and JL revised the manuscript. WH, RZ, and SJ collected data. All co-authors participated in the discussion of the results.

### Conflict of Interest Statement

The authors declare that the research was conducted in the absence of any commercial or financial relationships that could be construed as a potential conflict of interest.
